# Fluorographene based Ultrasensitive Ammonia Sensor

**DOI:** 10.1038/srep25221

**Published:** 2016-05-04

**Authors:** Kiran Kumar Tadi, Shubhadeep Pal, Tharangattu N. Narayanan

**Affiliations:** 1 TIFR-Centre for Interdisciplinary Sciences, Tata Institute of Fundamental Research, Hyderabad – 500075, India

## Abstract

Single molecule detection using graphene can be brought by tuning the interactions *via* specific dopants. Electrostatic interaction between the most electronegative element fluorine (F) and hydrogen (H) is one of the strong interactions in hydrogen bonding, and here we report the selective binding of ammonia/ammonium with F in fluorographene (FG) resulting to a change in the impedance of the system. Very low limit of detection value of ~0.44 pM with linearity over wide range of concentrations (1 pM–0.1 μM) is achieved using the FG based impedance sensor, andthisscreen printed FG sensor works in both ionized (ammonium) and un-ionized ammonia sensing platforms. The interaction energies of FG and NH_3_/NH_4_^+^ are evaluated using density functional theory calculations and the interactions are mapped. Here FGs with two different amounts of fluorinecontents −~5 atomic% (C_39_H_16_F_2_) and ~24 atomic% (C_39_H_16_F_12_) - are theoretically and experimentally studied for selective, high sensitive and ultra-low level detection of ammonia. Fast responding, high sensitive, large area patternable FG based sensor platform demonstrated here can open new avenues for the development of point-of-care devices and clinical sensors.

Several articles have been reported in the past discussing the development of various gas sensors, and ammonia is one of the gases taken seriously due to its natural and industrial origin^1^,^2^,^3^,^4^,^5^. But low level detection of ammonia (<2 ppb for environmental monitoring and <50 ppb for breath analysis) is still lacking a state of the art detection method, and its development is highly desirable for the development of clinical ammonia sensors^1^. Low dosages of ammonia can cause problems to human respiratory, skin, eyes etc., and its prolonged exposure can leads to pulmonary oedema^2^. Since ammonia is also excreted from human body in the form of urea, breathe ammonia measurement (measuring the quantity in the exhaled gas) can be a diagnostic tool for monitoring the function of kidney or ulcers caused by *Helicobacter pylori*bacterial stomach infection. The amount of exhaled air available will be minimal and hence high sensitive ammonia breathe sensors are highly demanding, but lacking at present. Apart from ammonia gas sensors, analysis of ammonia (ionized/un-ionized) level in blood is also of interest in medicine. Blood ammonia monitoring has of interests in sports medicines (0.1 to 10 ppm) and pediatrics (>100 μmol/L) too^3^,^4^,^5^.

Presently various sensing platforms are existing for ammonia: metal oxide based sensors^6^,^7^,^8^ (by conductance change due to the chemisorption of gas molecule), catalytic ammonia sensors (by catalytic activity of certain metals towards ammonia gas and the ammonia concentration dependency in the charge carriers)^9^,^10^, conducting polymer based ammonia sensors (by a two-fold oxidation-reduction mechanism)^11^,^12^, optical and spectrometric ammonia detection (cause in the coloration up on ammonia exposure as a measure)^13^, gas permeable membranes based selective detection techniques etc. are the most frequently used techniques^14^,^15^. But these methods lack the synergy of selective, sensitive, cost effective, and fast detection platform. A clinical ammonia sensor demands the above mentioned features along with a limit of detection value (LOD) of ~50 ppb within a response time of a few minutes. Existing selective optical methods are inadequate for the development of a portable (point of care (POCs)) ammonia sensors where limited sample needs to be detected using economically viable routes^1^.

Graphene based biosensors are receiving tremendous scientific attention due to the engineering possibilities of graphene by bringing specificity and sensitivity in the device *via* doping and defects. Further, robustness and availabilities of different detections mechanisms of the graphene based devices (electronic transducers (field effect transistor), electrochemical (conductometry, potentiometric, amperometric, and impedance) sensors etc.) attract the development of graphene based sensors^16^,^17^,^18^. Electrostatic interaction can bring specificity in the binding of molecules to graphene and hence graphene based sensors are capable of detecting individual gas molecules^19^. Charge transfer kinetics between graphene and adsorbed molecules can be tuned by doping, and hence the response time of a graphene based sensor can also be engineered. An external molecule can change the carrier density of graphene surface and itmayalso act as chemically doped entity on graphene driven by the electrostatic interactions among them^20^. Heteroatom doping on graphene can enhance the catalytic activity of the neighborhood of the dopant, and this can enhance the adsorption of molecules and affect the conductivity of graphene backbone. Moreover, it is also important to study the interaction between adsorbed molecules and graphene to understand the charge transfer mechanism, and tailoring the response by defects/dopants^20^.

Tang *et al.* have reported Density Functional Theory (DFT) calculations based graphene oxide (GO) – ammonia interactions^21^. Adsorption of ammonia on GO is stronger than graphene due to the presence of active functional groups such as hydroxyl and epoxy – where they form hydrogen bonding with ammonia leading to a charge transfer process. But the poor stability of GO in various solutions^22^, low thermal stability, instability of GO under long exposure of ammonia (possible reduction), and difficulties in controlling/benchmarking the extend of oxidation (C/O ratio) are still bottlenecks in the development of GO based gas sensors. Moreover, GO is an electrical insulator and also less electrochemically active. Recently, Ghosh *et al.* reported a reduced GO (RGO) based conductometric ammonia sensor with the LOD of 200ppm and also mentioned its some extend of selectivity towards ammonia^23^. The detection mechanism of RGO sensor is same as that in GO with an enhanced electrical conduction platform due to the reduction of some functional groups. Hence RGO cannot be a recommendable sensing platform for ammonia sensing. But, hydrogen bonding possibilities of ammonia with other thermally and chemically stable functional groups of graphene may be a viable option for graphene based ammonia sensor. Hydrogen bonding between the most electronegative element fluorine (F) and ammonia will be an ideal option, and hence fluorographene with optimum fluorine content (to optimize the electrical conductivity) can selectively detect ammonia with very high sensitivity.

Relatively high thermal stability of fluorographene (300–400 °C)^24^ will help the sustainability of FG based sensors. In the recent past, one of the authors has extensively studied the properties of FG derived from fluorinated graphite polymer ((CF_0.25_)_x_)^25^,^26^,^27^,^28^. It was found that fluorination alters the physical, chemical, electrical and electrochemical properties of graphene/GO. The high polarity of C-F bond even modifies the dielectric permittivity of graphene and it also induce sparamagnetism to graphene/GO backbone^26^. The charge transfer studies on fluorinated graphene systems indicate that the density of states near the Fermi level can also be altered by the fluorine doping^27^. Hence it is established that FG is stable even after the exposure to various alkaline/acid environments and also after elevated heating (100 °C).

In the present study, we report the interactions of FG and ammonia (unionized and ionized (ammonium)) using DFT calculations. The interactions between graphene and ammonia are also explored to compare the strength of FG-ammonia interactions. The FGs in the present study are derived from fluorinated graphene oxide (FGO, with 2-types of FGOs −5 at.% fluorine and 24~ at.% fluorine) and the role of residual functional groups in the interactions is not studied. Later, using Fourier Transform-Infrared (FT-IR) studies, we found that the interaction between fluorine and ammonia is prominent than other hydrogen bonding interactions *via* residual groups. FG based screen printed electrode is developed and its efficacy in ammonia sensing using impedance spectroscopy is demonstrated. Large area patternability of chemically derived FG is shown using a soft-lithography process, indicating the possibilities of FG based different sensor geometries.

## Results and Discussions

### Interaction Energy Calculations

**Figure 1:**
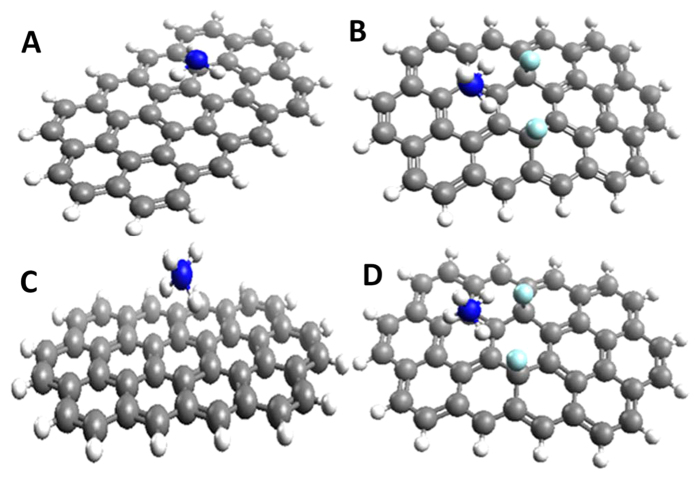
Optimized graphene/FG and ammonia/ammonium structures: (**A**) graphene-ammonia (C_39_H_16_-NH_3_), (**B**) FG -ammonia (C_39_H_16_F_2_-NH_3_), (**C**) graphene-ammonium (C_39_H_16_-NH_4_^+^), and (**D**) FG– ammonium (C_39_H_16_F_2_-NH_4_^+^).

FG has a carbon honeycomb lattice like in graphene with covalent fluorine (F) attached to carbon *via* sp^3^ hybridization. FG is an atomically layered material as it is evident from the electron microscope images given in the supporting information, Figure S1A,B. The bond lengths were calculated from fully optimized structures of graphene and FG (~5at%) and the values are given in the supporting information, Table S1. It is evident from the Table S1 that the edge bond lengths of C-C and C=C are less than the internal bond lengths of the same, and it is due to the strains at the edges, as reported by other researchers^29^. Table 1 lists the basis set superposition error (BSSE) corrected interaction energies of graphene and FG complexes with ammonia and ammonium ion (NH_4_^+^). The DFT studies reveal that FG is having strong interactions (−14.15 kcal/mol)with ammonia compared to graphene (−1.29 kcal/mol) due to the strong hydrogen bonding between C-F in FG and N-H in ammonia. The optimized structures of complexes are shown in Fig. 1. From the optimized structures, it is found that the N-H groups of ammonia are interacting with fluorine atoms present in the FG. The simulated closest distance of approach between FG and ammonia is about 2.90 Å whereas in case of graphene, the distance is found to be 3.10 Å. Moreover, an increase in the C-F bond length of FG from 1.416 Å to 1.426 Å is also observed after the complex formation with ammonia, and it is later verified using Fourier Transform Infrared spectroscopy (FT-IR) studies. These results indicate the presence of weak H-bonding (result of a dipolar electrostatic interaction) involved between the N-H group of ammonia and fluorine of FG. In the case of ammonium ion, even though the interaction energy of FG-NH_4_^+^ (−53.72 kcal/mol) is higher than the G-NH_4_^+^, the difference is less than that in the case of ammonia. This might be due to the enhanced electrostatic interaction between positively charged of ammonium ion and graphene (π electrons).

**Figure 2:**
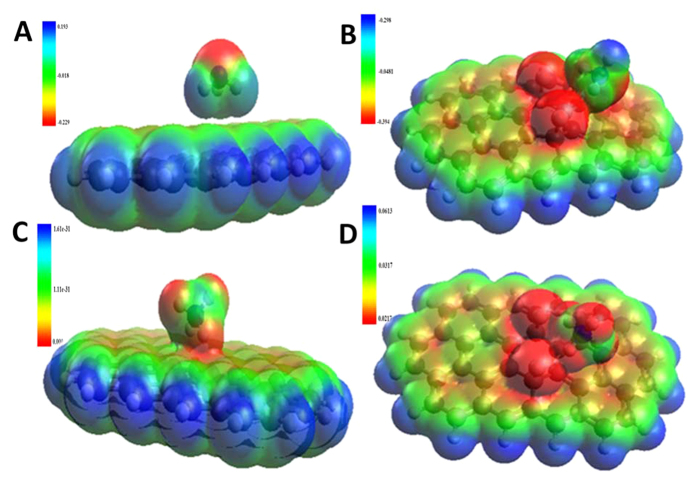
Electrostatic potential plots for (**A**) graphene-ammonia, (**B**) FG-ammonia, (**C**) graphene-ammonium, and (**D**) FG-ammonium.

The optimized structures and electrostatic interactions between ammonia/ionized ammonia (NH_4_^+^) and graphene/FG were calculated from HOMO and LUMO energies of the corresponding complexes. The calculated energy gaps are shown in Table 2. It is evident from the Table 2 that the energy gaps between NH_3_/NH_4_^+^ and FG is less than that of NH_3_/NH_4_^+^ and graphene. This can be due to the strong interactions of NH_3_ or NH_4_^+^ with FG, making the energy differences between HOMO and LUMO smaller. Further, the electrostatic potential maps of the interactions are shown in Fig. 2. The positive charged 1s orbital of hydrogen on NH_3_/NH_4_^+^ has strong electrostatic interaction with negatively charged 2p orbital of fluorine on FG, leading to a hydrogen bonding (electrostatic) interaction. The interaction maps show considerable orbital overlapping of NH_3_ and NH_4_^+^ with FG, while little interaction between NH_3_ and graphene is observed as it is reported by other researchers^30^. But NH_4_^+^ shows a better interaction with graphene due to its positive charge.

### FT-IR and micro Raman analysis

**Figure 3:**
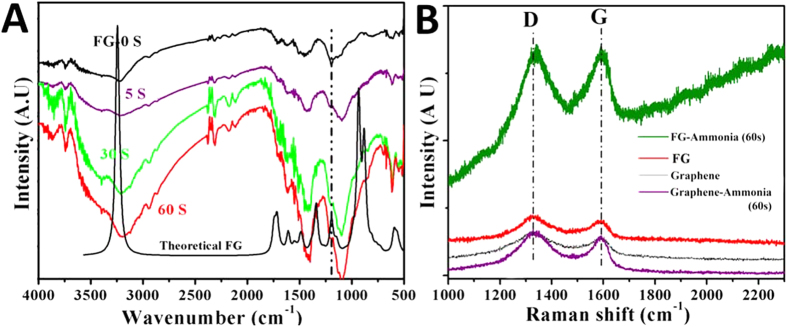
(**A**) FT-IR spectra of pristine FG (theoretical (y-axis is absorbance) and experimental (FG 0S, y-axis transmittance)) and after exposure of ammonia gas (at a pressure ~1psi). (**B**) Raman spectra (632 nm excitation).

The theoretical prediction of interaction between FG and ammonia is experimentally validated using FT-IR and micro Raman spectroscopy. Chemically derived (explained in the method section) FG powder (low resolution transmission electron microscope (TEM) image and high angle annular dark field TEM images are shown in supporting information Figures S1A,B, indicate the crystallinity and two dimensional sheet like morphology of FG) having 5 at. % F content (calculated using X-ray photoelectron spectroscopy (XPS), the C1s spectrum of FG is given in supporting information Figure S1C) is used for the investigation. The FT-IR spectrum of FG is also simulated and is shown in Fig. 3A (y-axis of theoretical curve is absorbance), and it exactly matches with that of FG (experimental)^25^,^26^. The presence of covalent C-F linkages in FG is further confirmed from the F^19^ Nuclear Magnetic Resonance (NMR) spectrum, as shown in the supporting information, Figure S1D. It has to be noted that the absorption peak at ~3250 cm^−1^ in theoretical FG is due to the =CH stretching of the end terminated hydrogen while in the case of FG experimental spectrum it is due to the –OH stretching from residual functional groups. In Fig. 3A, FG-0s depicts the experimental FTIR spectrum (y-axis is % transmittance unlike the theoretical spectrum) of FG without ammonia exposure. The covalent C-F peak at ~1200 cm^−1^is clear in both theoretical and experimental spectra. The FG has been exposed to ammonia gas (pressure ~1 psi) for different exposure times, and the corresponding FT-IR spectra are shown in Fig. 3A. The increase in exposure time increases the intensity of C-F vibrations along with a continuous red shift in the vibration frequency. This increase in the intensity of C-F vibration along with the red shift is the characteristic feature of normal hydrogen bonding^31^. It is also noticed that vibration frequency bands corresponding to residual –OH stretching or other oxygen functionalities (residual) of FG are not changed after the exposure of ammonia. This indicates that ammonia has a preferential binding with fluorine than with other functional groups.

The micro-Raman spectra of graphene and FG before and after exposing to ammonia are shown in Fig. 3B. All the spectra show typical graphitic Raman signatures corresponding to G and D peaks at 1600 and 1350 cm^−1^, respectively. The G band of graphene originates from the vibration of in-plane sp^2^ carbon. The D band is attributed to the defects in graphene. There are no considerable changes in the positions and intensity ratios of D and G bands before and after the exposure of ammonia indicating that the graphene structure is not disturbed after the ammonia exposure (structure distortion or reduction of oxygen functionalities *via* ammonia exposure is well reported the literature)^22^.

### Impedance Sensor

**Figure 4:**
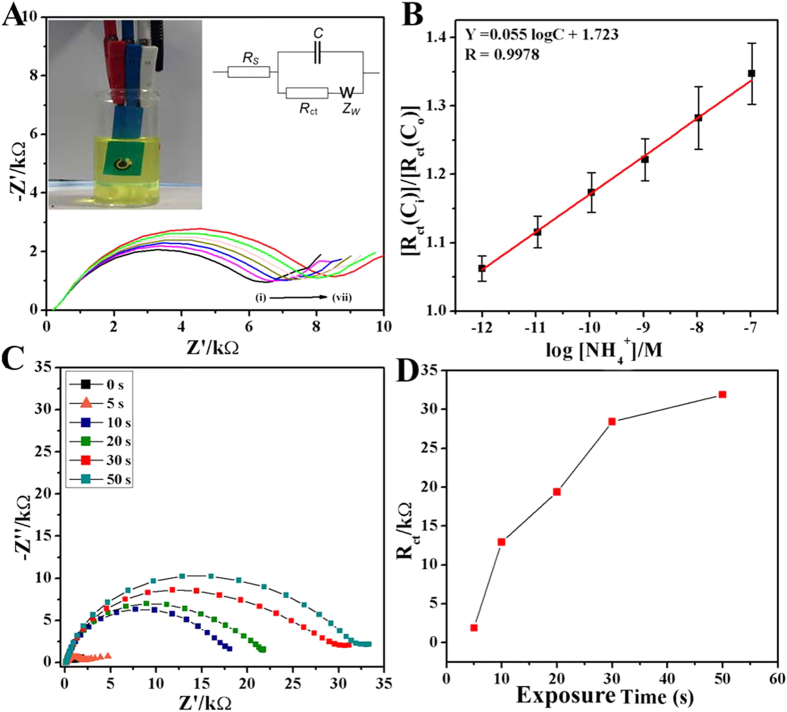
(**A**) Nyquist plots of FG coated electrode on SPE sensor for varying ammonium ion (NH_4_^+^) concentrations (i. blank, ii. 1 pM, iii. 10 pM, iv. 100 pM, v. 1 nM, vi. 10 nM, and vii. 0.1 μM), (inset) the photograph of an FG coated SPE sensor, (**B**) normalized charge transfer resistance for various ammonium ion concentrations, (**C**) Nyquist plots showing increased impedance with increase in direct NH_3_ exposing time, (**D**) R_ct_ values with varying direct NH_3_ exposures.

The above mentioned selective interaction of ammonia/ammonium with fluorine in FG *via* the electrostatic hydrogen bonding is utilized for the development of an ammonia sensor, as explained in the following section. Electrochemical impedance spectroscopy (EIS) was used to study the binding of ammonia on FG modified screen printed electrodes (Figure S2). The Fig. 4A illustrates the Nyquist plots, graph between the real (Z′) and imaginary (−Z′′) parts of impedance, using the screen printed electrode (SPE, shown in Fig. 4, and supporting information – where working electrode is modified with graphene samples as explained in the method section) obtained after the gradual increase in concentration of ammonia (ionized ammonia) from 1 pM to 0.1 μM (i–vii).

It is evident from the Fig. 4A that the charge transfer resistance (R_ct_) increases with increase in ammonium ion concentration. The increase in R_ct_ is attributed to the binding of ammonium to the FG* via* hydrogen bonding. In order to confirm that the observed change in impedance is due to surface modification of SPE and not due to superimposed effects, ratio of charge transfer resistance for the desired concentration (R_ct_(C_i_)) and charge transfer resistance of the blank FG electrode (R_ct_(C_o_)) is plotted against the logarithm of ammonia concentration (Fig. 4B). Figure 4B can be linearly fitted tothe following equation; R_ct_(C_i_)/R_ct_(C_o_) = 1.723 + 0.055 log C_NH3_. This shows that the sensor works in a linear concentration of ammonia in the range of 1 pM to 0.1 μM with a correlation coefficient of 0.998. Sensitivity of the sensor can be deduced from slope of this curve, resulting to a value of ~0.055 M^−1^. The limit of detection (LOD) of the sensor has been calculated according to Long and Wineforder method^32^:


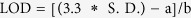


where S.D. is the standard deviation of the blank, a is the linear coefficient, and b is the angular coefficient (sensitivity). The calculated LOD is found to be ~0.44pM.

**Figure 5:**
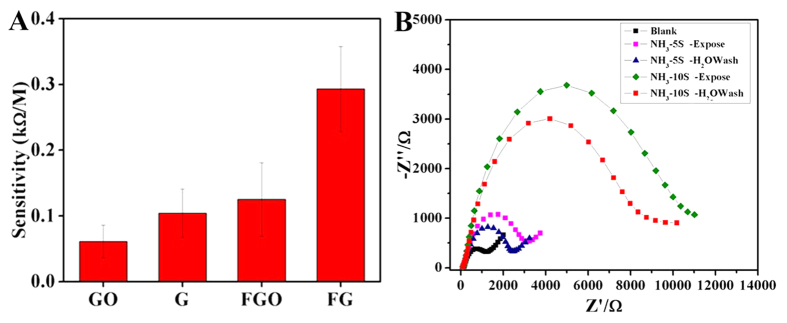
(**A**) Sensitivity of various electrodes towards ammonium ion (NH_4_^+^) sensing, (**B**) reversibility of FG-NH_3_ sensor.

Relative change in EIS data is more reliable for sensing applications than absolute impedance. Previous discussion indicates that a graph between the change in R_ct_ (ΔR_ct_) values and the logarithm of ammonia concentrationsreveals a linear detection range for ammonia concentrations in the range of 1 pM to 0.1 μM with 4.4 × 10^−13^M (0.44 pM) limit of detection. The linear relationship could be characterized using the linear equation: ΔR_ct_ (kΩ) = 3.832 + 0.293 log C_NH3_ (kΩ). This FG modified SPE exhibits a sensitivity of 0.293 kΩ M^−1^ with a correlation coefficient of 0.997 and standard deviation of 0.018 kΩ (Fig. 5A). A curve between logarithm of frequency and imaginary part of impedance (−Z”) has also been plotted (supplementary information, Figure S3A). Figure S3A shows that −Z” increases continuously with increase in ammonia concentration.

Further, EIS measurements are also conducted by direct purging of ammonia in to the phosphate buffer solution (PBS) (10 mM, pH 7.0) containing the mixture of 5 mM[Fe(CN)_6_]^4−^ (ferrocyanide) and 5 mM[Fe(CN)_6_]^3−^ (ferricyanide). The experimental set up is shown in supporting information Figure S4. The impedance spectra (supporting information Figure S5) show a continuous increase in impedance with ammonia bubbling time. This also indicates that the interaction between FG and ammonia and the fast response in the sensing (within a few seconds).

In order to check the efficacy of FG based ammonia sensor for direct ammonia detection, ammonia gas has been directly exposed to the FG modified electrodes (<1 psi pressure, 7 mm^2^ area) and the EIS was conducted immediately after the exposure. The EIS spectra are recorded for different times of ammonia exposure as mentioned in the Fig. 4C, and the corresponding Nyquist plots are shown. The variation in R_ct_with exposure time seems to be linearly increased with the exposure of ammonia (Fig. 4D). The values of R_ct_are linearly increased up to 30 s and then the response becomes lessen.

Further the impedance response of the bare (SPE), graphene oxide (GO), graphene and FGO coated electrodes with increase in concentration of ammonia (ionized ammonia) is studied. Sensitivity calibration curves (concentrations of NH_4_^+^*vs*ΔR_ct_) over a range of ammonia concentration from 1 pM to 0.1 μM for the various modified electrodes are shown in Fig. 5A. From the plot, it is clear that the GO coated SPE has the lowest sensitivity towards ammonia (sensitivity 61 Ω M^−1^), while the sensitivity has been slightly increased to 104 Ω M^−1^ after the reduction of GO. FG showed the highest sensitivity (293 Ω M^−1^). However, the sensitivity of the FGO (125 Ω M^−1^) is less than that of FG, but higher than that of GO. It has been discussed in our previous reports that GO and FGO have similar structure, morphology and C/O ratio^27^. The only difference between them is the presence of fluorine (here ~5 at.%) in FGO. Hence this enhanced sensitivity of FG/FGO is due to the presence of fluorine in the honeycomb lattice.

Reversibility (whether the binding of analyte is reversible or not) of an electrochemical sensor is one of important features of a chemical sensor. In the present case, reversibility of the electrode has been checked by performing the impedance studies after direct exposing to NH_3_ gas (as discussed in the previous section) followed by a simple washing of electrode with deionized water (running water). The decrease in the impedance (R_ct_) after washing is evident from the Fig. 5B, indicating that the electrodes are reversible.

In order to study the effect of higher amount of fluorine doped graphene (C_39_H_16_F_12_) for ammonia sensing, DFT calculations were conducted on ~24 atomic % fluorine containing graphene (HFG) (the optimized structure is shown in supporting information, Figure S6). The BSSE corrected interaction energy for HFG-NH_3_ is found to be −3.926210211 kcal/mol. This indicates that HFG-NH_3_interactions are weaker than that of FG-NH_3_. The C-F bond lengths before and after NH_3_ stabilization are calculated as 1.378 Å and 1.380 Å respectively. These values are in agreement with the recent calculations of bond lengths of fluorgraphene^33^. Unlike in the case of FG, there is negligible change in C-F bond length of HFG after ammonia stabilization. Moreover, the closest distance between NH_3_ and HFG is found to be 3.76 Å, which is larger than that in FG (2.9 Å). This indicates that the binding of HFG with NH_3_is rather weak. The poor interaction between HFG and NH_3_ is further confirmed experimentally *via* EIS studies (supporting information, Figure S7). Here the change in impedance with NH_3_ concentration is found to be minimal. The increase in fluorination will increases the defect levels and also decreases the electrical conductivity^25^,^26^,^27^,^28^. Hence this study indicates that the amount of F in FG needs to be optimized for the development of a practical ammonia sensor. This optimization is important in applied aspects of other doped graphene too, where the enhanced defect levels and interactions can adversely affect the properties.

The added advantage of chemically derived FG is the ability to make micro-electrodes and patternable devices using simple techniques such as soft-lithography^34^. Large area electrode patterns constructed using FG are shown in supporting information Figure S8. Since soft-lithography (polydimethylsiloxane (PDMS) stamps) based imprinting (solvent assisted micro-molding technique, solvent used is dimethyl formamide (DMF)) can be made on substrates like plastics/glasses/cellulosic papers etc., sensors with flexibilities and visible light transmitting can be developed using these chemically derived atomic layers. A Raman mapping conducted on such an FG pattern is also shown in supporting figure S8 showing the uniformity of patterns. This indicates that the FG based ammonia sensors can bring features such as sensitivity, selectivity, fast response and reversibility along with other novel aspects of modern POCs and biosensors such as transparency and mechanical flexibility.

## Conclusions

This study reveals the possibility of making a commercial ammonia sensor (both gaseous and ionized) by doping graphene with appropriate amount of fluorine - bringing all the key features (sensitivity, selectivity, fast response and reversibility) required for a practical chemisensor. The interactions between ammonia (both ionized and un-ionized) and FG were studied using DFT calculations, and the results were compared with that of graphene-ammonia/ammonium ion systems. An augmented electrostatic interaction is observed in FG-ammonia/ammonium ion (−14.15 kcal/mol and −53.72 kcal/mol respectively) systemsthan that of graphene – ammonia/ammonium ion (−1.29 kcal/mol and −37.26 kcal/mol), and it is established through the hydrogen bonding interactions *via* fluorine and hydrogen. FG modified screen printed electrodes were studied for ammonia sensing in both solution and gas phases, and a very low limit of detection of 4.4 × 10^−13^M (0.44 pM) with linearity over a wide range of concentrations (1 pM–0.1 μM) is achieved using this FG based impedance sensing platform. This study also points out the importance of optimization of dopant levels in graphene for its best sensing performance. Further, the possibility of large scale microelectrode patterning ability using these chemically derived FG with the aid of soft-lithography is demonstrated and it reveals the promises of FG towards the development of flexible and transparent ammonia sensors and POCs. Recent research indicates that such a flexible impedance sensing device can be a futuristic *in-vivo* non-invasive diagnostic platform for static and dynamic continuous monitoring modes^16^,^35^.

## Methods

### Computational Methods

Thestructures of FGs and graphene, and the interactions were studied *via* Density Functional Theory calculations using Gaussian 09 soft-ware package^36^. All the structures were subjected to full geometry optimizations without any constraints at the M05-2X/6-31G*level. This method was chosen as it appears to be better compared to the more popular B3LYP alternatives when modeling non-bonding interaction^37^.

All stationary points were characterized as minima after verifying the presence of all real frequencies. Single point energy calculations were carried out at the M05-2X/6-311++G** level. The interaction energy (IE) was calculated using equation (2) as the difference between the total energy of the complex (E_G-X_ or E_FG-X_) and the sum of the total energy of the parent 2D-nano material (E_graphene_(E_G_) or E_FG_) and the binding molecule (E_X_, X = NH_3_ or NH_4_^+^).


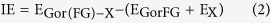


The interaction energy was corrected for basis set superposition error (BSSE) using the counter poise (CP) correction scheme (which is within 1 kcal/mol).

Frontier molecular orbitals of the complexes were studied using the energy level gap between the highest occupied molecular orbital (HOMO) and the lowest unoccupied molecular orbital (LUMO) respectively.

### Materials Synthesis and Characterization

Fluorinated graphite polymer (cat. No. 42537) was purchased from Alfa Aesar. Graphite polymer (particle size < 20 microns), ammonium chloride and KMnO_4_ were procured from Sigma-Aldrich. All chemicals were analytical reagent grade and used without further purification. Aqueous solutions were prepared using Millipore water received from Milli-Q system (Millipore Inc.).

The preparation of GO and FGO was adopted from the previous report^25^,^26^,^27^,^28^. A mixture of concentrated sulfuric acid and phosphoric acid in 9:1 ratio (360 mL: 40 mL) was added to graphite (3.0 g, 1 wt equiv.). It was followed by gradual flake by flake addition of KMnO_4_ (18.0 g, 6 wt equiv.). The reactants were then heated to 90 °C and stirred for 12 h. The reactants were brought it down to room temperature and poured onto ice with 30% H_2_O_2_ (3 mL) and stirred for 2 h. The obtained solid suspension was washed several times successively with excess of deionized water, 30%HCl, and ethanol. Finally, it was coagulated with 200 mL of ether and filtered over a PTFE membrane with a 0.22 μm pore size. Fluorinated GO (FGO) was also prepared in similar method by taking Fluorinated graphite polymer instead of graphite as the starting material^25^,^26^. Two different types of FGOs (having different fluorine content) were resulted from the phase separation of the material while H_2_O_2_ was added to the mixture. The reduction of functional groups in FGO results to the formation of FG and it was conducted using electrochemical reduction. The synthesized GO or FGO (4 mg) was well dispersed in 2 ml of deionized water by sonication for 2 hours. Then 5 μL of the solution was drop casted on a conductive electrode surface (e.g. SPE, carbon) and dried under ambient conditions to get GO or FGO coated SPE. GO or FGO was electrochemically reduced by CV scanning from 0.0 to −1.5 V in N_2_ purged 0.1 M pH 5.0 PBS (K_2_HPO_4_/KH_2_PO_4_) for 20 cycles, and then rinsed with water and dried at room temperature. For direct sensing of ammonia gas, GO or FGO was thermally reduced by placing at 90 °C for 3 h in vacuum oven.

Electrochemical impedance studies (EIS) were conducted using a Biologic potentiostat, model SP-300. Low-cost, screen-printed electrodes (schematic of the device is shown in supporting information, Figure S2) were procured from Zensor (Taiwan). These consist of a 3 mm diameter working electrode and an arc-shaped auxiliary electrode (both made of graphitic carbon powder) and a Ag/AgCl pellet reference electrode all on a 50 × 13 mm plastic substrate. The EIS measurements were also conducted in the 3-electrode system consists of glassy carbon electrode as working electrode Ag/AgCl (Sat. KCl) as reference electrode and spiral platinum wire as counter electrode. EIS measurements were carried out at half-wave peak potential of the redox mixture in PBS solution (10 mM, pH 7.0) containing a mixture of 5 mM [Fe(CN)_6_]^4−^ and 5 mM[Fe(CN)_6_]^3^ over the frequency range 10^5^–0.01 Hz with 5 mV as the alternating current amplitude. Using the redox probe (5 mM [Fe(CN)_6_]^3−/4−^), change in charge transfer resistance (R_ct_) at electrode/electrolyte interface has been investigated in electrochemicalimpedance.

FTIR analysis was conducted in transmittance mode on a Bruker (model: Alpha) spectrometer. The micro-Raman spectra of GO and FGO samples were studied using LabRamXploRA Raman spectroscope (excitement wavelength 632 nm).

### Supporting Information

TEM images of FG, XPS and NMR data of FG, schematics of screen printed electrode and ammonia sensing set up are provided in the supporting file. Theoretically evaluated bond lengths observed in free graphene and FG is also provided in the supporting file. SEM image and Raman mapping of large area printed FG microelectrodes is provided. Direct ammonia sensing data is also provided. Ammonia sensing results (theoretical and experimental) with high fluorine containing graphene is also provided. This material is available free of charge via the Internet at http://pubs.acs.org.

## Additional Information

**How to cite this article**: Tadi, K. K. *et al.* Fluorographene based Ultrasensitive Ammonia Sensor. *Sci. Rep.* **6**, 25221; doi: 10.1038/srep25221 (2016).

## Supplementary Material

Supplementary Information

## Figures and Tables

**Table 1:** Interaction energies of various complexes.

S. No	Molecule/Complex	Single Point Energy (Hartrees)	BSSE Corrected Interaction Energy	
			in Hartrees	in kcal/mol
1.	FG	−1733.97254		
2.	G	−1534.34988		
3.	NH_3_	−56.547803		
4.	FG-NH_3_	−1790.54289	−0.0225	−14.15
5.	Graphene-NH_3_	−1590.89964	−0.0019	−1.29
6.	FG-NH_4_^+^	−1790.945667	−0.0856	−53.72
7.	Graphene-NH_4_^+^	−1591.29678	−0.0594	−37.26

**Table 2:** HOMO-LUMO gaps for various complexes.

	Complex	HOMO (eV)	LUMO (eV)	Band-gap (eV)
1	FG-NH_3_	−0.08023	−0.20139	0.121
2	Graphene-NH_3_	−0.07987	−0.2022	0.122
3	FG-NH_4_^+^	−0.18358	−0.30316	0.119
4	Graphene-NH_4_^+^	−0.16822	−0.30455	0.136
