# Supplementary material for: Fluorographene based Ultrasensitive Ammonia Sensor
Source: Sci Rep. 2016 May 4;6:25221. doi: 10.1038/srep25221 (PMC4855224; doi:10.1038/srep25221)
Supplement: Supplementary Information [file srep25221-s1.pdf]

Supporting Information

Fluorographene based Ultrasensitive Ammonia Sensor

Kiran K. Tadi, Shubhadeep Pal, and Tharangattu N. Narayanan*

TIFR-Centre for Interdisciplinary Sciences, Tata Institute of Fundamental Research, Hyderabad – 500075, India.

(* Corresponding Author: tnn@tifrh.res.in or tn_narayanan@yahoo.com)

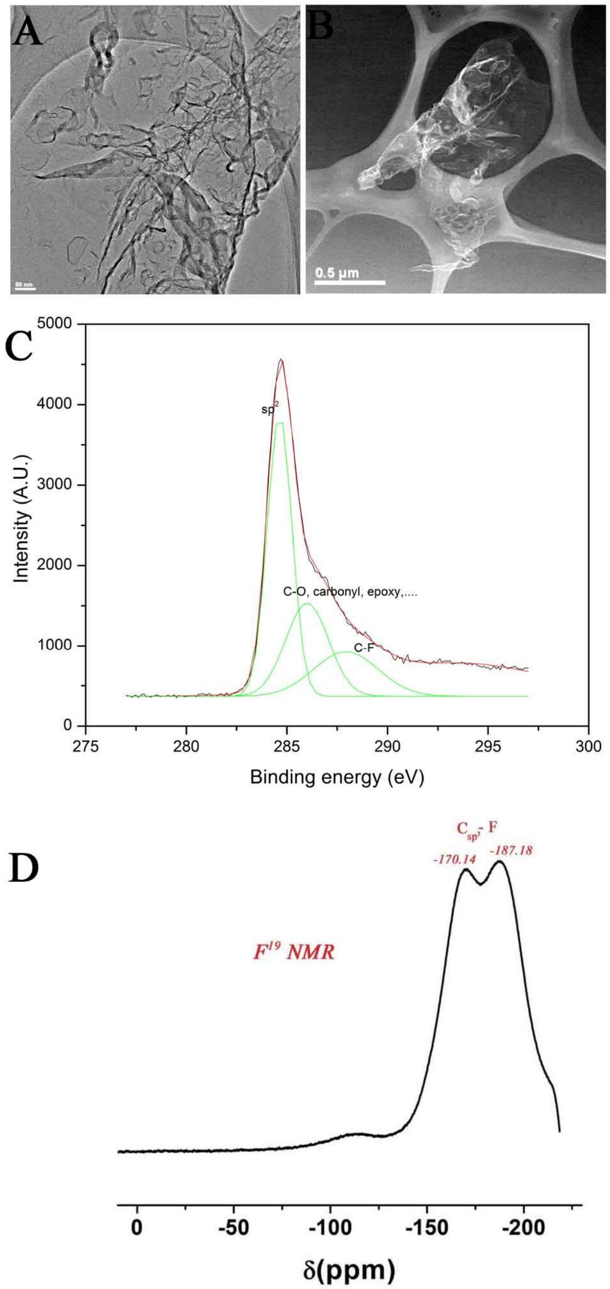

Figure S1. (A) Low resolution TEM image showing large area sheet like morphology of FG, (B) high angle annular dark field TEM image of FG, showing its crystallinity and transparent sheet like morphology. (C) C1S XPS spectrum of the FG showing the presence of C-F, C=C and residual oxygen functionalities (C-O), and (D) ^{19}F NMR spectrum of FG collected using Bruker FT Nuclear Magnetic Resonance (NMR) spectrometer (400 MHz). The spectrum shows peaks placed at -170.4 ppm and -187.18 ppm. The peaks placed closed to -180 ppm show the presence of covalent C-F bonds (C_{sp3}-F).

Table S1: Bond lengths observed in free graphene and FG molecules

Bond	Bond lengths in	
	graphene	FG
C-C$_{(internal)}$	1.41-1.43	1.37-1.42
C=C$_{(internal)}$	1.40-1.42	1.40-1.42
C-C$_{(edge)}$	1.38-1.41	1.35-1.41
C=C$_{(edge)}$	1.35-1.41	1.37-1.41
C-H	1.08	1.08
C-F	-	1.41

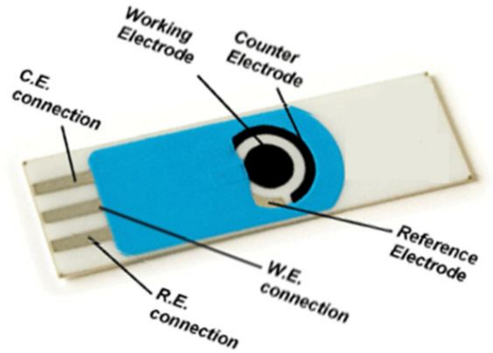

Figure S2: The structure of a commercial screen printed electrode.

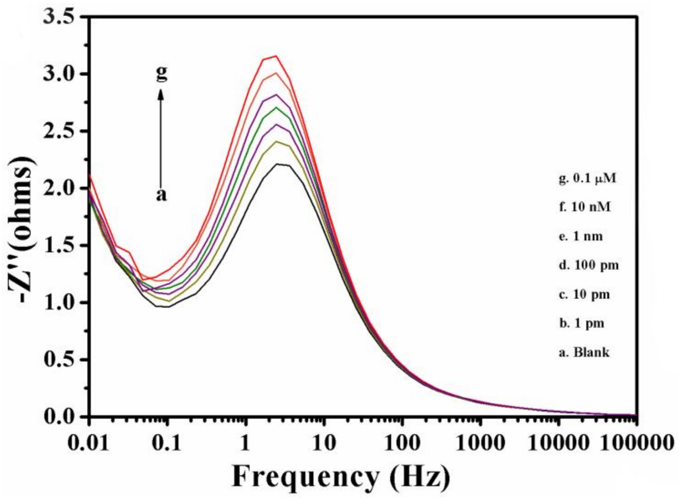

Figure S3: (A) EIS spectra of FG coated SPE for varying ammonium ion (NH_4^+) concentrations.

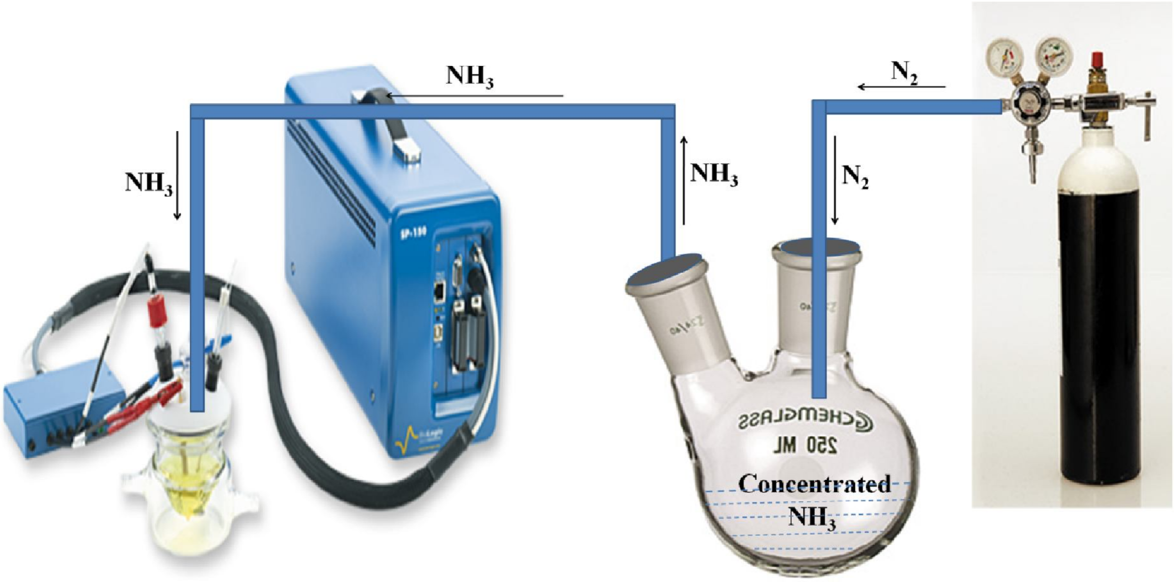

Figure S4: Schematic representation of bubbling of ammonia gas into the electrochemical cell using nitrogen as carrier gas (pressure<1 psi).

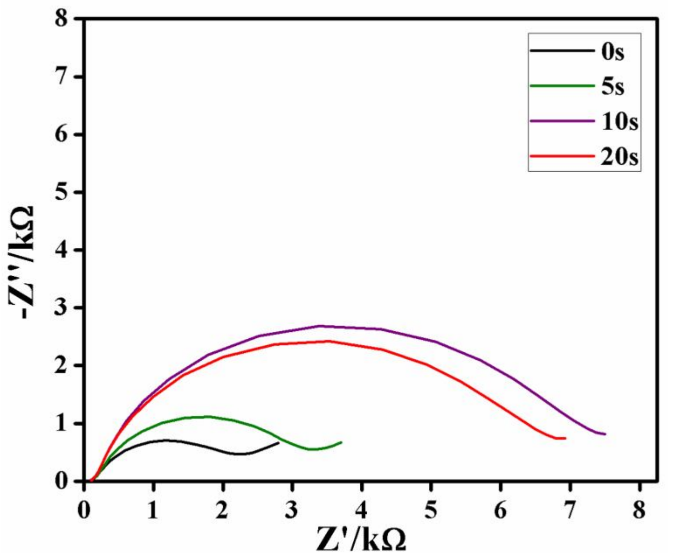

Figure S5: Nyquist plots showing increase in charge transfer resistance with increase in ammonia bubbling time.

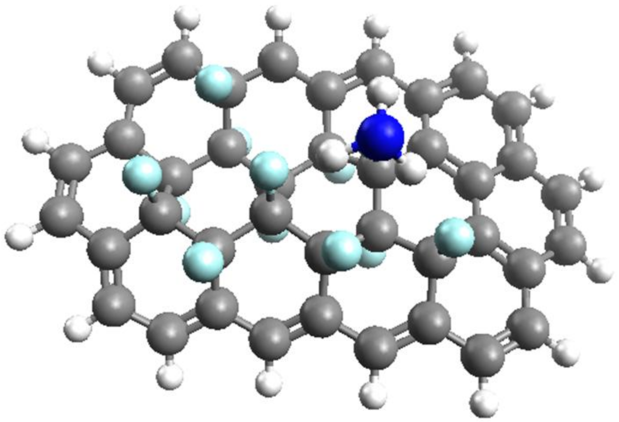

Figure S6: Optimized HFG-NH_3 structure.

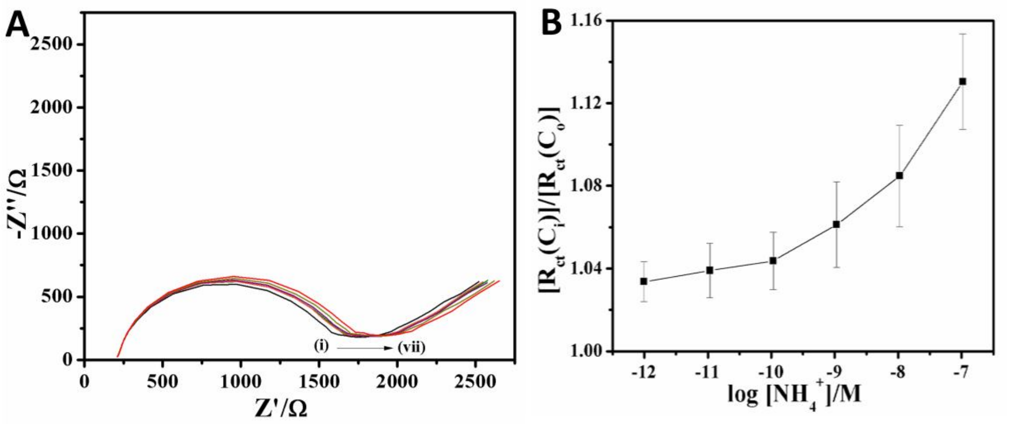

Figure S7. (A) Nyquist plots of HFG coated electrode on SPE sensor for varying ammonium ion (NH_4^+) concentrations (i. blank, ii. 1 pM, iii. 10 pM, iv. 100 pM, v. 1 nM, vi. 10 nM, vii. 0.1 μM) (B) normalized charge transfer resistance for various ammonium ion concentrations.

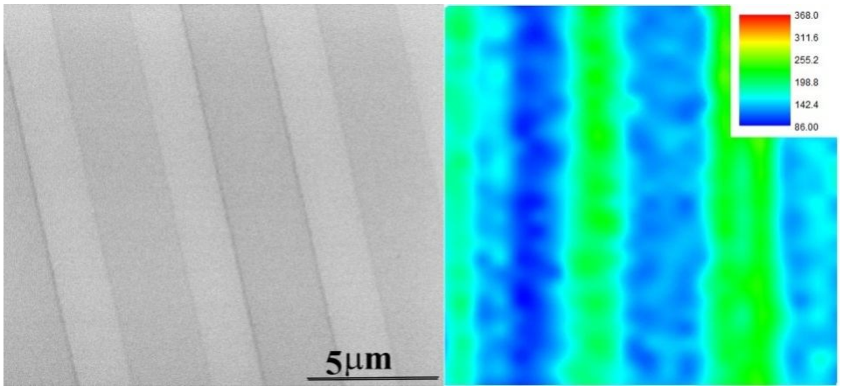

Figure S8: Scanning electron microscopic image of FG micro-electrodes printed in a silica substrate using PDMS pre-patterned stamp via soft-lithography technique. FG solution in DMF is infiltrated in to the stamp-base sandwich via nano capillarity and released the stamp after 24 hours. Two dimensional Raman (D band mapping) intensity mapping on the pattern indicates the formation of uniform strips (the scale indicates the relative intensity of the D-band).
